# Tuberculosis of prostate: a rare clinical image

**DOI:** 10.11604/pamj.2024.47.195.43119

**Published:** 2024-04-17

**Authors:** Shreya Giri Goswami, Kishor Hiwale

**Affiliations:** 1 Department of Pathology, Jawaharlal Nehru Medical College, Datta Meghe Institute of Higher Education and Research, Sawangi (Meghe), Wardha, Maharashtra, India

**Keywords:** Tuberculosis, Langhans giant cell, granuloma

## Image in medicine

Tuberculosis is a prevalent disease in India, primarily involving the lungs, lymph nodes, and abdomen. While it commonly involves the kidneys and epididymis in the genitourinary tract, occurrence in the prostate is extremely rare (2.6%). It raises suspicion towards benign hyperplasia and often bladder carcinoma, therefore often goes overlooked and undiagnosed. These lesions are often secondary. Early lesions are challenging to detect while advanced ones may result in an enlarged, fluctuant, and tender prostate on palpation. A 74-year-old male presented in the outpatient department of urology with complaints of increased frequency of micturition, poor streaming, and nocturia for 2 months. There was no history of constitutional symptoms such as fever, evening rise of temperature, or weight loss. The patient was hypertensive but did not give any history of tuberculosis in the past. The blood picture revealed no abnormalities. The patient was not immunocompromised. Ultrasonography revealed an enlarged prostate of size 28.9 gm with smooth outlines. Prostate-specific antigen (PSA) was 10.2 ng/ml. Prostatitis and benign hyperplasia were considered differentials. The patient underwent transurethral resection of the prostate (TURP) in view of the given differentials. The microscopic examination of the TURP chips revealed epitheloid granuloma. Caseous necrosis in between the prostate glands and stroma surrounded by Langhans giant cells and lymphocytes. The histopathological diagnosis of tubercular prostatitis was given. Chest X-ray was normal. Polymerase chain reaction (PCR) and Mantoux test were positive pointing towards tuberculosis. Antitubercular therapy (ATT) was started as per the national protocol.

**Figure 1:**
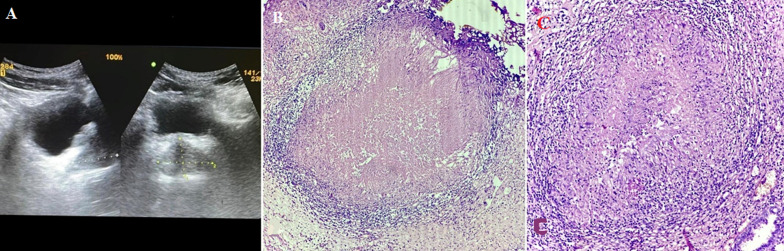
A) ultrasonography showing enlarged prostate; B) photomicrograph prostate: section shows caseous necrosis between the glands and stroma (Hematoxylin & Eosin, 10x); C) photomicrograph: section shows epitheloid granuloma surrounded by Langhans giant cells and lymphocytes (Hematoxylin & Eosin, 40x)

